# Contrasting Performance of Two Winter Wheat Varieties Susceptible to Leaf Rust under Diverse Pathogen Pressure, Fungicide Application, and Cultivation Practices

**DOI:** 10.3390/jof10060401

**Published:** 2024-06-02

**Authors:** Radivoje Jevtić, Vesna Župunski, Dragan Živančev, Emilija Arsov, Sasa Mitrev, Ljupco Mihajlov, Branka Orbović

**Affiliations:** 1 Institute of Field and Vegetable Crops, 21000 Novi Sad, Serbia; vesna.zupunski@ifvcns.ns.ac.rs (V.Ž.); dragan.zivancev@ifvcns.ns.ac.rs (D.Ž.); branka.orbovic@ifvcns.ns.ac.rs (B.O.); 2 Faculty of Agriculture, Goce Delcev University, 2000 Stip, North Macedonia; emilija.arsov@ugd.edu.mk (E.A.); sasa.mitrev@ugd.edu.mk (S.M.); ljupco.mihajlov@ugd.edu.mk (L.M.)

**Keywords:** yield, TKW, crude protein, leaf rust, cultivation practice, susceptibility, fungicide

## Abstract

This study investigated the relationship between yield, thousand kernel weight (TKW), and crude protein of soft white winter wheat–club variety (Barbee) and soft white winter wheat common variety (Zvezdana) susceptible to leaf rust and powdery mildew under different cultivation practices. Results revealed divergence in associations between yield, TKW, and crude protein loss of winter wheat varieties susceptible to obligate pathogens. Under the same level of leaf rust infection, N-input limited yield loss of the two varieties but not to the same extent. TKW loss was affected only by variety×cultivation practice and was significantly correlated with yield loss (r = −0.727, *p* = 0.011) and crude protein loss (r = −0.600, *p* = 0.05) only in club winter wheat. We suspected that Ninput affects the difference in the relationship between yield and TKW loss among varieties. Crude protein and yield loss had a low association (R^2^ = 18%, *p* = 0.05). Finally, this study indicated that more attention should be paid to the determination of pathogen pressure that triggers yield loss. It also pointed out that yield, TKW, and crude protein response to fungicides could differ in susceptible varieties. The contribution of fungicide to yield enhancement was highly associated with the specific reaction of the variety to pathogen infection rather than solely the disease level itself.

## 1. Introduction

Wheat is considered an important field crop since it provides more than 20% of the calorific intake for almost two-thirds of the human population and contributes to nearly 30% of the world’s grain production and 50% of the world grain trade [1]. The factors influencing yield, yield components, and quality parameters have been studied for decades, but the most influencing factors were analysed separately without consideration of their interactions [2,3]. Breeding high-yielding wheat varieties with good quality is not an easy task since these traits can be negatively correlated or not correlated at all [4,5].

One of the yield components associated with grain quality is thousand kernel weight (TKW). Higher TKW indicates better germination of wheat grain and better milling quality [6]. Although the significant effect of varieties, agro-ecological conditions, and diseases on TKW has been reported in previous studies [7,8], the difference in response of wheat varieties on the combined effect of interactions between disease indices and cultivation practices has rarely been taken into consideration [9]. The crude protein content is usually used as a minimum quality assurance check and is also proven to be highly influenced by climatic factors and cultivation practices [10,11]. However, the combined effect of wheat pathogens and cultivation practices on quality parameters is rarely reported [9].

In addition to powdery mildew (*Blumeria graminis* f. sp. *tritici*), rust diseases (*Puccinia graminis* f. sp. *tritici*, *Puccinia triticina*, *Puccinia striiformis* f. sp. *tritici*) are the most important obligate pathogens of wheat [12]. The reduction in winter wheat yields caused by powdery mildew could reach 45% [13]. Leaf rust (*P. triticina*) was the prevalent rust species in Serbia until 2014 when yellow rust (*P. striiformis* f. sp. *tritici*) predominated [14]. The predominance of yellow rust in Serbia was also recorded in 2018, while in the rest of the years, co-occurrence patterns of these two diseases were observed [14]. Yield losses caused by leaf rust were as high as 50%, but they also reached 60% under yellow rust infection [14]. 

According to U.S. statute, there are three subclasses of soft white wheat: “Soft White Wheat”, “White Club Wheat”, and “Western White Wheat” [15]. Head (spike) morphology defines features of the first two subclasses. Club wheats are characterised by the reduced overall length of the spike since they possess the “club” or C locus that reduces the internode length of the rachis. Soft white wheat is referred to as “common soft white” since it does not possess the C locus, which results in a “common” spike. The C locus influences kernel size and shape and does not have any direct bearing on the quality of club wheat. In contrast to that, the quality of club wheat varieties is defined by breeding and selection [15]. Although “common” and “club” winter wheat share a number of similarities, they can be distinguished by grain and flour parameters and distinctly different end-use qualities [15]. Consequently, it could be expected that these two classes of wheat react differently to the same cultivation practice, climatic conditions, and pathogen attack. 

Zwer et al. [16] investigated differences in spike characteristics, yield, and yield components of common and club wheat varieties and indicated that club wheat had greater adaptive potential than common wheat in areas with marginal moisture. Fewer moisture requirements for seed germination, faster germination, strong culms, and firm spikes made club wheat more adaptive to arid agronomic zones when compared to common wheat. Although resistant to drought with outstanding quality and resistance to common and some races of dwarf bunt, the production of early club wheat varieties such as Omar decreased after the major stripe rust epidemic of 1960 in the Pacific Northwest. Afterward, a multiline approach utilising a mixture of lines with different resistance genes to stripe rust provided durable resistance to stripe rust in newer varieties of club wheat [17]. The advantage of club wheat over common wheat with respect to resistance to *F. culmorum* and *F. graminearum* causing root rot is also associated with the better resistance of club wheat to water stress [18]. 

Nowadays, pest control is challenging since wheat pathogens continuously change under selection pressures of agro-ecological conditions, resistant varieties, and applied pesticides. Rust fungi are highly adaptive to new resistance genes and are also highly genetically diverse for races [12,19]. In addition, Jevtić et al. [14] indicated that genotypes and their reaction to climatic elements in certain phenological stages could have a strong impact on interactions among obligate pathogens and their predominance. Although the disease development of obligate pathogens and their impacts on yield and quality performance are greater in very susceptible and susceptible varieties, it has also been reported that the yield loss and disease index of obligate pathogens are not always as closely correlated as expected in susceptible varieties. The effects of cultivation practices on these differences are still understated in studies [20]. Current varieties are generally adapted to high-input systems, but there are conflicting reports on whether elite varieties adapted for optimal cropping conditions have good performance under suboptimal environments [21].

In addition to diverse susceptibility, the variability in yield responses of varieties to obligate pathogens is also associated with seasonal weather fluctuations, yield potential, and timing of infection [22]. Early-season infections by powdery mildew can significantly diminish yield in susceptible varieties, lowering it by up to 25% [22]. During the early stages of crop growth, infection with leaf rust could lead to more than a 50% loss of production [23]. Powdery mildew affects a decrease in photosynthetic leaf area, the depletion of crop-available nutrients, and, critically, hampers yield potential via excessive tiller production that fails to yield heads. Severe powdery mildew infections during initial growth phases can also stunt plant growth and delay maturity, amplifying the risk of reinfection. Early infection by leaf rust results in weak plants and poor root and tiller development [22]. Leaf rust causes the most damage when severe rusting covers the upper leaves before flowering. Early defoliation can also occur, reducing the time for grain fill and leading to the creation of smaller kernels. Later-season infections with powdery mildew, occurring between stem elongation and flowering, diminish photosynthetic leaf area, leading to diminished grain size, decreased yields, and quality deterioration. The earlier the onset of infection, the more prolonged its persistence and the greater its spread up the plant, intensifying potential yield losses [22]. 

Ploughing was considered to be a promising cultivation practice in reducing the level of disease inoculum [24,25,26], even for foliar diseases that can overwinter in the stubble from the previous crop, such as powdery mildew (*Blumeria graminis* f. sp. *tritici)* and stem rust (*P. graminis* f. sp. *tritici)* [27]. Nowadays, the choice of cultivation practice that will be applied in wheat growing areas depends on the complexity of agro-ecological conditions and socioeconomic aspects, but there is a still lack of knowledge on factors that could jeopardise the expected effects of tillage practices [28]. The effectiveness of fungicide application in controlling powdery mildew and leaf rust is also highly associated with factors that impact their life cycles, infection potentials, and interactions with the host plant. Understanding these differences makes preconditions for more effective disease management strategies [22].

Knowing that breeding of varieties with advantageous genetic traits for low input is often overlooked and that the regulatory network for plant responses to abiotic and biotic stress consists of many mechanisms and components that may function antagonistically [29,30,31], it was hypothesised in this study that susceptible wheat varieties could react differently on infection of obligate pathogens when cultivated under different cultivation practices (monoculture with reduced N input and conventional N input with crop rotation). Consequently, the main objectives of this study were to measure (1) the effect cultivation practices of susceptible varieties belonging to different wheat classes and their association with yield, thousand kernel weight (TKW), crude protein content, and disease indexes (leaf rust and powdery mildew); (2) the difference in the relationship between yield, TKW, and crude protein response to fungicide application of “club” (Barbee) and “common” (Zvezdana) winter wheat varieties susceptible to powdery mildew and leaf rust. The Barbee is a bearded, semi-dwarf soft white winter wheat—club variety (*Triticum aestivum compactum*), and Zvezdana is soft white winter wheat—common variety (*Triticum aestivum aestivum*).

## 2. Materials and Methods

Data were obtained from research and development trials (R&D) on fungicide efficacy against obligate pathogens (*Blumeria* and *Puccinia*). Fungicide efficacy trials were conducted in 2021 in the municipality of Novi Sad (Vojvodina, northern province of Serbia) under the direction of the Institute of Field and Vegetable Crops, Novi Sad, Serbia. All permissions for trial conductance were defined by contracts with chemical companies. Research also complied with relevant institutional, national, and international regulations. The trials were carried out at two locations differing in cultivation practices: (1) monoculture + reduced N input and (2) conventional N input with crop rotation. The trials included fungicide-treated and fungicide-untreated plots at both locations, and fungicides were applied in the same manner. More details on model varieties, conditions of trial setup, and assessments of disease levels, yield, TKW, crude protein, and oil content of soft white winter wheat—club variety (Barbee) and soft white winter wheat—common variety (Zvezdana) under naturally occurring inoculum of obligate pathogens are given below.

### 2.1. Model Varieties

In this study, we used two varieties susceptible to powdery mildew [32] and leaf rust [33] but belonging to different wheat classes. The variety Barbee is a bearded, semi-dwarf soft white winter wheat–club (*Triticum aestivum* ssp. *compactum*). It was originated by USDA-ARS and Washington State University and was released in 1976. Its flour quality is suited for pastries, cookies, and other white wheat products but not for bread. It was developed for production in Idaho and Washington state (USA) and was expected to be cultivated instead of variety Paha in areas where stripe races jeopardised wheat production. Barbee is mid-season in maturity. On a scale of 1 (low or poor) to 10 (high or good), Barbee is indexed at 8 for straw strength, 5 for emergence, 5 for winter hardiness, and 5 for test weight. Concerning disease resistance, Barbee is resistant to common bunt, moderately susceptible to dwarf bunt, resistant to stripe rust, and moderately resistant to flag smut. It was also used as a susceptible check in field trials in many countries because of its high susceptibility to leaf rust [33] and powdery mildew [32,34,35]. In the study of Jevtić et al. [20] the DIs of leaf rust in Barbee ranged from 22% to 68% in untreated plots during the period from 2006 to 2013 at the locality Rimski šančevi (Serbia). DIs of powdery mildew in the same study ranged from 21% to 30%. 

Zvezdana is a soft white winter wheat–common (*Triticum aestivum* ssp. *aestivum*). It was released by the Institute of Field and Vegetable Crops, Serbia, in 2005, and it is still commercially available. Zvezdana is high-yielding and resistant to lodging. Zvezdana is characterised as a medium–early cultivar with very good winter hardiness. Stem height has a range from 75 to 83 cm, the test weight is 80–84 kg, and it belongs to the A1-A2 Farinograph quality class (according to the Hungarian Standard method 6369/653 (MSZ 1988) [36]). In Germany, wheat varieties are classified into four classes based on quality parameters: E-class, i.e., elite quality; A-class, i.e., A-quality; B-class, i.e., bread making, and C-class, i.e., used for stock-feed purposes https://www.bundessortenamt.de (accessed on 13 March 2023). Parameters that are usually tested for quality criteria are protein content, grain hardness, sedimentation values, falling number, wet gluten content, dough rheology, etc. Zvezdana is also reported as being highly susceptible to variation in climatic factors during the growing season [37].

Zwer et al. [16] examined variances in spike characteristics, yield, and yield components between common and club wheat varieties. They suggested that club wheat exhibited superior adaptive capabilities over common wheat in regions with limited moisture. Club wheat required less moisture for seed germination, displayed quicker germination, and possessed robust culms and firm spikes, rendering it more suitable for arid agricultural zones compared to common wheat. These findings are also supported by Bafus et al. [38], who reported that club wheat can be the highest yielding variety in Central Oregon during years with temperature extremes, especially when fertilised equally to soft wheat with 350 lb/acre of 30-10-0-7. 

### 2.2. Field Trial

Fungicide efficacy trials were conducted under naturally occurring inoculum of obligate pathogens and under the same climatic conditions but using different cultivation practices: (1) 20-year monoculture + reduced N input; and (2) long-term crop rotation + conventional N input. Monoculture and crop rotation were applied in order to provide different levels of pathogen pressure. Reduced N input included the usage of 68 kg of nitrogen per ha, while conventional N input cultivation included the usage of 100 kg of nitrogen per ha. Long-term crop rotation included a 4-year crop rotation design that is applied for decades. 

The soil type in both localities was a slightly carbonated loamy chernozem. The soil properties include a predominantly loamy and crumbly structure with stable aggregates and a neutral to slightly alkaline reaction. Field trials were arranged in a randomised block design comprising four replicates. The plot size of each replicate was 10 m^2^. The trials included 10 fungicide-sprayed and non-sprayed check treatments. 

All fungicides were applied independently in BBCH 51–59 (inflorescence emergence, heading) and also in combination in three growth stages: BBCH 32–33 (node 2 at least 2 cm above node 1; node 3 at least 2 cm above node 2); BBCH 39 (flag leaf stage: flag leaf fully unrolled, ligule just visible) and BBCH 61–65 (beginning of flowering: first anthers visible; full flowering: 50% of anthers mature). Fungicides were applied in the same manner at both locations, which differed in cultivation practices and the amount of nitrogen applied. Since contracts with chemical companies do not allow preliminary publication of the data, the trade names of tested fungicides are not provided in this manuscript. Active ingredients used in the study were prothioconazole, spiroxamine, trifloxystrobin, bixafen, fluopyram, mefentrifluconazole, fluksapiroksad, pyraclostrobin, and benzovindiflupyr. Fungicides were applied using calibrated field crop sprayers with fan nozzles at 300 kPa pressure and 200 L of water per hectare. The sowing date for winter wheat was 20 October (optimal time of sowing), and the harvest date was 30 June.

### 2.3. Climatic Conditions

The majority of the territory of the Republic of Serbia is under the Cfb climate type according to the Köppen–Geiger Climate Classification. It is characterised as a warm temperate–fully humid climate type with warm summers, but from 1961 until the present, a significant increase in temperature change and in precipitation patterns has been observed [39]. In the year 2021, when the trial was conducted, lower total precipitation than the eight-year average was recorded in the time of the flowering and grain-filling period (Table 1).

### 2.4. Disease Assessments

Assessments of leaf disease severity were made using a modified Cobb’s scale [40] at the growth stage 71–73 BBCH (kernel watery; early milk), known to be highly related to yield [41]. The disease indices (DI %) of leaf rust and powdery mildew were calculated by taking into consideration disease incidence and average disease severity according to [7] as follows:

DI (%) = [sum (class frequency × score of rating class)]/[(total number of plants) × (maximal disease index)] × 100

### 2.5. Yield 

The yield was measured for each plot at 15% water content. Yield loss (%) was determined as yield reduction in untreated plots compared with yield response to fungicide treatment, which provided the best control of wheat diseases (Equation (1)). Yield losses are determined for untreated plots at each locality differing in cultivation practices.

(1) $$Y(\%)=\left(\left(Y_{1}-Y_{2}\right)/Y_{1}\right)\times 100$$

*Y*_1_—the highest grain yield after fungicide treatment

*Y*_2_—grain yield of the non-sprayed check treatment

Yield response to fungicide application (%) using the following formula (Equation (2))

(2) $$Y(\%)=\left(\left(Y_{1}-Y_{2}\right)/Y_{2}\right)\times 100$$

*Y*_1_—grain yield of fungicide treatment for the best wheat disease control

*Y*_2_—grain yield of the non-sprayed check treatment

### 2.6. Crude Protein and Oil Content Assessments 

Crude protein and oil content were determined by handheld GrainSense NIR Analyzer (Oulu, Finland) and expressed as a percentage of dry matter. According to the study of Živančev et al. [42], the correlation between the standard Kjeldahl digestion method for protein determination and the handheld GrainSense NIR Analyzer was positive (r = 0.951). This device is the first of its kind in the world since it weighs only 820 g without batteries. The time of measuring is only three seconds, and the required sample size is between 60 and 80 kernels. It is based on near-infrared (NIR) technology with a 360° light penetration method (integrating sphere). 

### 2.7. Statistical Methods

The effects of variety, locality (differing in cultivation practices), variety × locality and the disease index of leaf rust × variety on yield, TKW, crude protein and oil content were examined using multiple regression modelling. Regression modelling was also applied to test the most influencing factors on yield, TKW, and crude protein loss in untreated plots as well as yield, TKW, and crude protein responses to fungicide application. In all regression models, the disease index of leaf rust was used as a continuous predictor, while variety (Barbee, Zvezdana) and locality (20-year monoculture + reduced N input and long-term crop rotation + conventional N input) were used as categorical predictors. Stepwise regression was applied since abiotic and biotic factors could be correlated not just to the yield, TKW, and crude protein loss/gain but also to each other (multicollinearity). Multicollinearity is addressed in this study because it can increase the variance of the regression coefficients. We also used VIFs in regression models to detect multicollinearity. The VIFs measure how much the variance of an estimated regression coefficient increases if predictors are correlated and will be 1 if there is no correlation between factors. In our study, VIFs did not exceed 1.2. The alpha level to enter and the alpha level to remove the influencing factors in the stepwise multiple regression were set by default to 0.15. Bursac et al. [43] reported that in stepwise multiple regression, an alpha level of 0.05 to enter and to remove the influencing factors could fail to identify important variables. 

Knowing that PCA requires quantitative and MCA qualitative variables only, associations between qualitative variables (variety, cultivation practice) and quantitative variables (yield loss, TKW loss, crude protein loss, disease index of leaf rust) were analysed using principal correspondence analysis with mixed data (PCAmix). PCAmix includes ordinary principal component analysis (PCA) and multiple correspondence analysis (MCA) as special cases.

To provide more information on which means are significantly different and to estimate how much they differ, we used a general linear model and Tukey’s pairwise comparisons with 95% confidence. The correlation between variables was analysed using Spearman’s coefficient of correlation.

The analysis was performed using Minitab 17 Statistical Software (trial version) and XLSTAT 2022.5.1 (1392) in Microsoft Excel 16.0.17425 [44]. Visualisation of PCAmix analysis was performed using Package ‘ggplot2’ in RStudio software (RStudio 2021.09.2+382 “Ghost Orchid” Release) [45].

## 3. Results

On average, when both fungicide-treated and fungicide-untreated plots were taken into consideration under both types of cultivation, yield (8.6 t/ha), TKW (31.8 g), and oil content (1.7%) of Barbee were significantly lower (*p* < 0.001) than yield (10.3 t/ha), TKW (43.0 g), and oil content (2.1%) of Zvezdana. However, the average crude protein of Barbee (11.7%) was significantly higher (*p* < 0.001) than that of Zvezdana (10.1%). 

**Figure 1:**
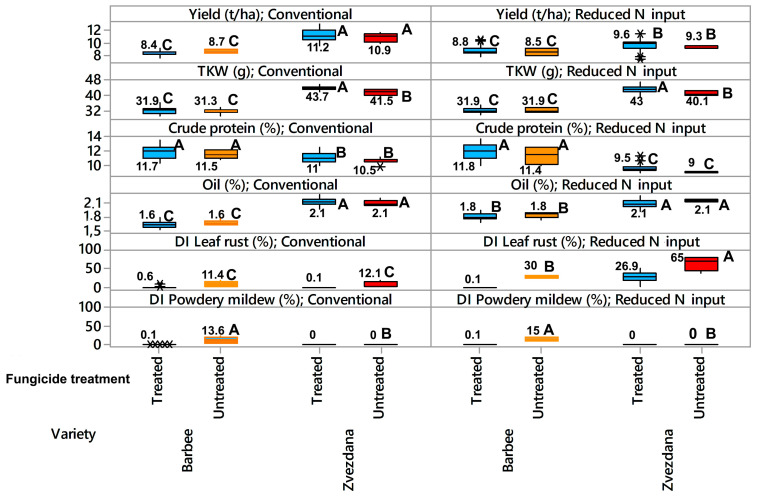
Yield, TKW, crude protein, oil content, disease index of leaf rust, and disease index of powdery mildew in “club” winter wheat Barbee and “common” winter wheat Zvezdana in fungicide-treated (blue color) and fungicide-untreated plots (orange color for Barbee and red color for Zvezdana), cultivated under monoculture with reduced N input and crop rotation with conventional production. Tukey’s method was used to create confidence intervals for all pairwise differences between factor level means. The traits designated with different letters are significantly different. Sign “*” is outlier data label.

The average yield and crude protein content of Zvezdana were significantly higher when cultivated under conventional nitrogen input with crop rotation than under monoculture with reduced nitrogen input. In contrast, the yield and crude protein content of Barbee did not differ when grown under monoculture with reduced nitrogen input and conventional nitrogen input with crop rotation (Figure 1). The oil content of Zvezdana was significantly higher than that of Barbee and did not differ between the two cultivation practices. The oil content of Barbee was significantly affected by different cultivation systems. Thousand kernel weights (TKWs) were significantly different between the varieties. Cultivation practices did not affect the TKW of a single variety, but fungicide treatment did, only in the variety Zvezdana. Consequently, yield, crude protein, and oil content were significantly influenced by variety, locality (differing in cultivation practices), and the interaction between variety and locality. TKW was significantly influenced only by variety (*p* < 0.001) without interaction with cultivation practices (Table 2). 

Barbee and Zvezdana are both varieties susceptible to leaf rust and powdery mildew, but their reactions to obligate pathogens differed in two cultivation systems. Since we intended to measure how cultivation practices, differing in N input and previous crop rotation, affected infection levels of naturally occurring leaf rust and powdery mildew, we evaluated the effects of two cultivation systems on leaf rust and powdery mildew occurrence in untreated plots. In untreated plots, the disease index (DI) of leaf rust was greater under monoculture with reduced N input for both Zvezdana (65%) and Barbee (30%) than under conventional N input with crop rotation, where the DI of leaf rust for Zvezdana and Barbee were 12% and 11%, respectively. Regression modelling indicated that variety (*p* < 0.001), locality (*p* < 0.001), and the interaction between variety and locality (*p* < 0.001) influenced the DI of leaf rust (Table 2). Powdery mildew infected only Barbee, and the average disease index did not exceed 15% (monoculture with reduced N input) and 13.6% (conventional N input with crop rotation). Since the DI of powdery mildew in Barbee under two types of cultivation was not significantly different and did not significantly affect the yield and quality properties of Barbee in untreated plots (Table 2), the association of the DI of powdery mildew with cultivation practices, yield, TKW, and crude protein was not further analysed. 

**Figure 2:**
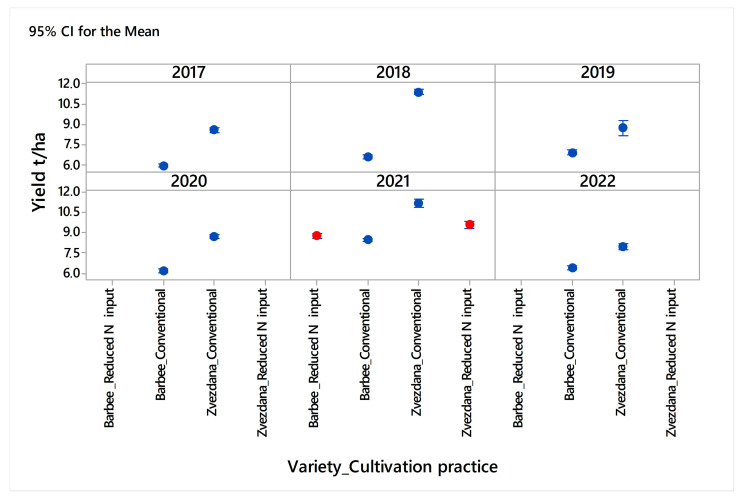
The yield relationship between common winter wheat Zvezdana and club winter wheat Barbee over a six-year period (2017–2022) in the locality of Rimski šančevi under conventional cultivation practice (blue marks). Red marks indicate yield reactions of varieties Barbee and Zvezdana when grown under monoculture with reduced N input.

It has to be pointed out that the average yields of fungicide-treated and fungicide-untreated plots of variety Barbee grown in the same location and under conventional N input with crop rotation were constantly lower than average yields of variety Zvezdana over the six-year period (from 2017 to 2022) (Figure 2). Consequently, variability in climatic conditions among the years was not expected to affect the yield relationship between Zvezdana and Barbee. Since the oil content is a parameter that is associated with germ size [46], and in the study of Moore et al. [46] did not correlate with grain yield, its association with yield, TKW, and crude protein was not further analysed, too.

### 3.1. Association between Losses in Yield, Thousand Kernel Weight (TKW), and Crude Protein Content, along with Leaf Rust Disease Index and Cultivation Practices in Barbee and Zvezdana Belonging to Different Wheat Classes 

In order to compare the reaction of susceptible varieties to leaf rust while grown under different cultivation practices (monoculture + reduced-N-input) and (crop rotation + conventional N-input), the yield, TKW, and crude protein losses were estimated in plots where fungicide treatments were not applied. It is important to notice that comparing only average absolute values of yield, TKW, and crude protein of two varieties in fungicide-treated and fungicide-untreated plots under the same type of cultivation will not produce exact information on how yield/TKW/crude protein in untreated plots changed with respect to the highest values of yield/TKW/crude protein obtained after fungicide treatment, providing the best control of wheat diseases. Thus, relative values in the form of losses of yield, TKW, and crude protein are used in further analysis instead of absolute values. 

**Figure 3:**
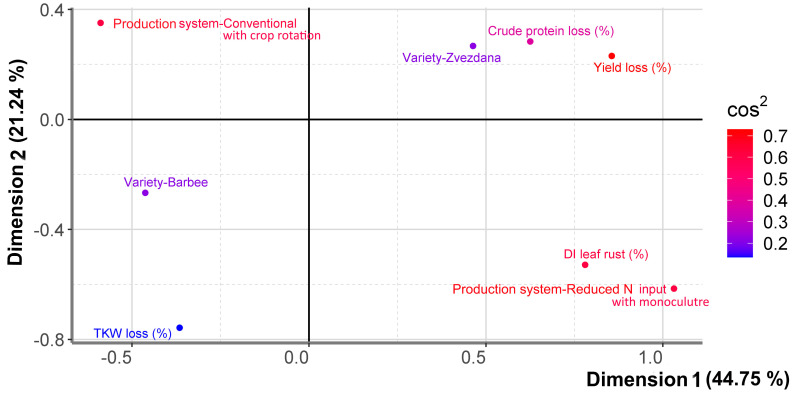
Graphical representation of PCAmix analysis between yield loss, TKW loss, crude protein loss, disease index of leaf rust, production system, and varieties differing in wheat classes.

PCAmix was performed to visualise a global pattern within the data and their associations. The first two dimensions contributed 65.99% to the overall variability (Figure 3). Overall, different production practices (reduced N input with monoculture and conventional N input with crop rotation) grew on opposite sides of the plot origin and contributed differently to the first two dimensions. Zvezdana and Barbee were also positioned on opposite sides of the plot origin. Yield and crude protein loss were not grouped together with TKW loss on a factor map, indicating that they had been affected by different factors. Yield and crude protein loss were more associated with the first dimension, while TKW loss was more associated with the second one. The disease index of leaf rust was grouped with monoculture growing with reduced N input, but its effect on yield, crude protein, and TKW loss of susceptible varieties was not straightforward and was positioned on the opposite side from both varieties. The quality of association between variable categories and a particular axis was presented with squared cosine (cos^2^). Squared cosine showed that yield loss, disease index of leaf rust, and production systems had the highest degree of association with the first dimension (red colour), while the variety and TKW had the lowest association with the first two dimensions (blue colour) (Figure 3). Crude protein loss was moderately associated with the first two dimensions. Regression analysis indicated that differences in crude protein loss between varieties and localities were non-significant in statistical terms, although variety Zvezdana suffered higher crude protein loss under monoculture with reduced N-input cultivation when compared with variety Barbee (Table S1).

Since yield loss, disease index of leaf rust, and cultivation practices had the highest contribution to the first dimension of PCAmix analysis and were also differently associated with Zvezdana and Barbee, their relationship is further presented in more detail.

#### 3.1.1. The Effect of Cultivation Practices on Difference in Yield Loss of Barbee and Zvezdana Varieties under Diverse Levels of Leaf Rust Infection 

Regression analysis of influencing factors on yield loss of two susceptible varieties was performed with the data originating from untreated plots and indicated that the most influencing factors on yield loss were variety (*p* = 0.013), locality (*p* < 0.001) and interaction variety × locality (*p* = 0.007) (Table S1). The coefficient of determination for the regression model was 69.9%. 

**Figure 4:**
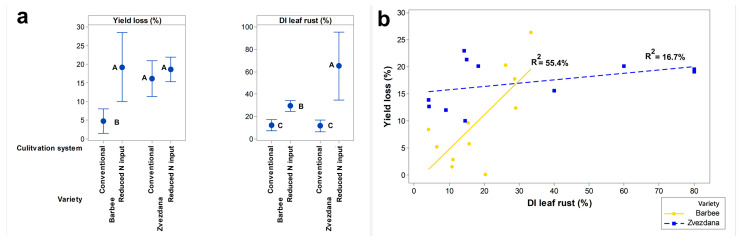
Yield loss of susceptible varieties under diverse levels of leaf rust infection and cultivation practices. (**a**) Interval plots show difference in yield loss and disease index of leaf rust in “club” winter wheat Barbee and “common” winter wheat Zvezdana cultivated under monoculture with reduced N input and crop rotation with conventional N input. (**b**) Linear regression of disease index of leaf rust on yield loss in “club” winter wheat Barbee and “common” winter wheat Zvezdana cultivated under monoculture with reduced N input and conventional production. The data originated from the plots where fungicides were not applied. Tukey’s method is used to create confidence intervals for all pairwise differences between factor level means. The traits designated with different letters are significantly different.

Although Barbee and Zvezdana are both varieties susceptible to leaf rust, their reaction to the pathogen pressure in a single locality under the same cultivation system was not the same. When cultivated under a conventional production system (crop rotation + conventional N input), the average yield loss of variety Barbee (4.7%) was significantly lower than that of variety Zvezdana (16.1%), although the average disease index of leaf rust on both Barbee (11.9%) and Zvezdana (11.3%) was not significantly different (Figure 4a). 

Under monoculture with reduced N-input farming without crop rotation, the average disease index (DI) of leaf rust for variety Barbee (29.3%) and variety Zvezdana (65%) was higher than that under the conventional production system. However, the higher DIs of leaf rust did not result in higher yield losses in both varieties compared to yield losses under lower pathogen pressure and conventional N input. The yield loss of variety Barbee under monoculture with reduced N input (19.2%) was significantly higher than the yield loss (4.7%) under conventional N input. However, variety Zvezdana did not exhibit significantly different yield losses when grown under lower pathogen pressure and reduced N input (18.6%) compared to higher pathogen pressure with conventional N input (16.1%) (Figure 4a). Spearman’s coefficient of correlation also showed a moderately significant positive correlation between yield loss and the disease index of leaf rust only in variety Barbee (r = 0.636, *p* = 0.035). After regression modelling, a significantly higher association between the disease index of leaf rust and yield loss was observed in variety Barbee (*p* = 0.009), contrary to variety Zvezdana (Figure 4b). Moreover, there was no significant correlation between these two traits in variety Zvezdana (*p* = 0.212). 

Since diverse levels of leaf rust infection and different levels of N input did not cause differences in yield losses of variety Zvezdana, the climatic conditions during flowering and grain filling were also considered as additional factors highly influencing yield loss of variety Zvezdana. The total rainfall during flowering and grain filling in 2021 (62.9 mm and 23.9 mm) was less than the eight-year averages (110.5 mm and 85.8 mm) (Table 1). Consequently, it is plausible to suppose that dry conditions during flowering and grain filling affected the yield performance of variety Zvezdana more than that of variety Barbee. Significantly higher yield losses of variety Barbee when grown under higher leaf rust infection and reduced nitrogen input in monoculture, compared to conventional nitrogen input with lower levels of pathogen pressure, indicated that the yield performance of variety Barbee was more affected by these factors rather than extreme climatic conditions.

#### 3.1.2. The Difference in the Relationship between Losses in Yield, Thousand Kernel Weight (TKW), and Crude Protein Content of ‘Club’ and ‘Common’ Winter Wheat Varieties Susceptible to Leaf Rust 

Since PCA is mostly used as a tool in exploratory data analysis, the relationship between losses of yield, TKW, and crude protein of two varieties belonging to different wheat classes was also analysed with regression modelling (Table S1). 

Regression modelling showed that factors influencing yield and TKW loss are not the same. In contrast to yield loss, which was significantly affected by variety (*p* = 0.013), locality (*p* < 0.001), and interaction variety × locality (*p* = 0.007), TKW loss was significantly affected only by interaction variety × locality (*p* = 0.021) (Table S1). In general, when both susceptible varieties were taken into consideration, Spearman’s coefficient of correlation between losses of TKW and yield was moderately significantly negative (r = −0.455, *p* = 0.034). However, when individual varieties were tested, the correlation between TKW loss and yield loss was moderately significantly negative only in the Barbee variety, while it was not significant in Zvezdana (Table 3). TKW loss of variety Barbee under cultivation with conventional N input (13.3%) was higher than that under reduced N input (8.6%), but according to the Tukey test, these differences were not statistically significantly different from those of variety Zvezdana under conventional N input (9.4%) and reduced N input (13.1%). Interestingly, Barbee and Zvezdana suffered higher TKW losses under different types of cultivation: Barbee under conventional N input with crop rotation and Zvezdana under reduced N input with monoculture. 

The association between crude protein loss and yield loss was low (R^2^ = 18%, *p* = 0.05) (Figure S1) but showed a similar trend in both Barbee and Zvezdana. The low association between yield and crude protein losses indicated that different factors influenced these two traits. Regression analysis showed that the effect of the disease index of leaf rust on crude protein loss (*p* = 0.148) should not be understated, although it was not significant at an alpha level of 0.05.

The association between crude protein loss and TKW loss was also different in the two varieties. Spearman’s coefficient of correlation indicated a significant moderate negative correlation between TKW loss and crude protein loss only in the variety Barbee (Table 3). 

### 3.2. The Difference in the Relationship between Yield, TKW, and Crude Protein Response to Fungicide Application of “Club” and “Common” Winter Wheat Varieties Susceptible to Leaf Rust 

In our study, fungicides were applied independently at BBCH 51–59 (inflorescence emergence, heading) and also in combination at three growth stages: BBCH 32–33, BBCH 39, and BBCH 61–65. The disease index (DI) of leaf rust on the Barbee variety, when all combinations of fungicide treatments were considered, averaged 2.5% under conventional nitrogen input with crop rotation and 3% under reduced nitrogen input with monoculture at BBCH 73–75. For the Zvezdana variety, when all fungicide treatments were considered, the average DI of leaf rust was 0.4% under conventional nitrogen input with crop rotation and 25% under reduced nitrogen input with monoculture at BBCH 73–75. 

**Figure 5:**
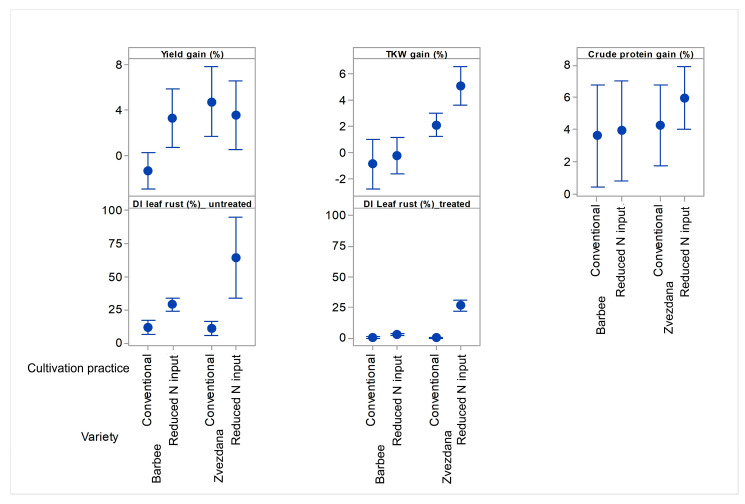
Averages of yield, TKW, crude protein, and disease index of leaf rust responses to fungicide treatment in “club” winter wheat Barbee and “common” winter wheat Zvezdana cultivated under monoculture with reduced N-input farming and crop rotation with conventional N input. Tukey’s method is used to create confidence intervals for all pairwise differences between factor level means.

When grown under conventional N input with crop rotation, the yield gain of Zvezdana after fungicide application (4.7%) was significantly higher than the average yield response to fungicide application of the Barbee variety (−1.3%) (Figure 5). At the same time, in untreated plots, leaf rust infection of Zvezdana and Barbee did not exceed 12%, and DIs were not significantly different. The greater contribution of fungicide treatment to yield gain in variety Zvezdana than in variety Barbee under the same level of pathogen pressure and cultivation practice suggests that the effect of fungicide treatment on yield response is variety-specific and not associated only with pathogen pressure and the ability of a fungicide to prevent or stop the development of pathogen infection but also with the reaction of varieties to environmental factors. Contrary to variety Barbee, Zvezdana is highly susceptible to unfavourable climatic conditions during flowering and grain filling, which, together with pathogen infection, affected yield loss in untreated plots and yield gain in treated ones. This result indicates that fungicide treatments can have a beneficial effect on yield response even under a low level of leaf rust infection, especially for varieties that are more susceptible to environmental stressors, such as variety Zvezdana. Variety Barbee suffered a low level of yield loss in untreated plots under conventional N input with crop rotation, and fungicide treatment had no effects on its yield response in treated ones. 

When varieties were grown under reduced nitrogen input and monoculture, the disease indices (DIs) of leaf rust in fungicide-untreated plots reached 29.3% in Barbee and 65% in Zvezdana, significantly higher than those in untreated plots under conventional nitrogen input with crop rotation. In fungicide-treated plots, the average DI of leaf rust was 3% in Barbee and 25% in Zvezdana. Nevertheless, yield gain after fungicide application was not significantly different between the varieties, equalling 3.3% in Barbee and 3.6% in Zvezdana. Interestingly, the yield gain in Barbee was significantly higher under conventional cultivation, while it remained at the same level of significance under two types of cultivation in Zvezdana (Figure 5). This suggests that the higher pathogen pressure of leaf rust and reduced nitrogen input with monoculture cultivation affected yield performance in Barbee more directly, making their relationship more correlated than in Zvezdana. 

The same level of yield gain obtained under diverse levels of leaf rust pressure and also under different types of cultivation in a single variety (Zvezdana) indicated that there is an infection level that triggers yield loss for each variety and is associated with its susceptibility not only to the pathogen itself but also to other environmental factors. Barbee exhibited a positive response to fungicide application when disease pressure in untreated plots led to DIs of leaf rust of 29.3%, while in Zvezdana, yield gain was expressed when DIs of leaf rust reached 11.3%. 

Contrary to the yield response of Zvezdana to fungicide treatment, the TKW achievements of Zvezdana in fungicide-treated plots were more influenced by the different types of cultivation. TKW gain in Zvezdana was more prominent under monoculture with reduced N-input conditions than under conventional N input (Figure 5). However, there was no difference in the TKW response of the Barbee variety to fungicide treatment under the two types of cultivation. It indicated that fungicide treatments contributed differently to yield and TKW responses in two susceptible varieties, which was also proven with regression modelling (Table S2). Regression analysis showed that interaction between fungicide treatment × disease index of leaf rust (*p* = 0.009) and variety × locality (*p* < 0.001) influenced yield response in treated plots. However, the effect of treatment × disease index of leaf rust (*p* = 0.104) and variety × locality (*p* = 0.140) on TKW response had less significance than the effect of variety (*p* < 0.001) and locality (*p* = 0.001) (Table S2).

Crude protein response to fungicide treatments was recorded in both varieties in the range from 3.8% (Barbee) to 5.2% (Zvezdana), but their differences were not statistically significant due to the broad range of confidence intervals (Figure 5). The broad range of confidence intervals for yield and crude protein responses in both varieties resulted from different contributions of 10 fungicide treatments and their combinations on these two traits. In this study, crude protein response to a fungicide application was significantly influenced only by leaf rust (*p* = 0.067) (Table S2). 

Interestingly, although within the same cultivar (Barbee), the association between thousand kernel weight (TKW) loss and crude protein loss was more pronounced than the association of TKW and crude protein gains. In Barbee, TKW loss and crude protein loss exhibited a high correlation (r = −0.727, *p* = 0.011), whereas the correlation between TKW and crude protein responses to fungicide application was lower (r = −0.367, *p* = 0.002) (Table 3 and Table 4). In Zvezdana, these two traits showed no correlation at all. 

## 4. Discussion

This study investigated the relationship between yield, thousand kernel weight (TKW), and crude protein of winter wheat varieties susceptible to obligate pathogens under different cultivation practices and the same climatic conditions. It was found that each variety exhibited a specific threshold of pathogen pressure that triggered yield loss and that the reaction of susceptible varieties to the same level of pathogen infection, in the same locality and under the same cultivation system, was not the same. Additionally, it was observed that susceptible varieties highly susceptible to unfavourable climatic conditions could exhibit the same level of yield loss under a broad range of pathogen pressures and a variable nitrogen supply. Differences in the associations among losses in yield, TKW, and crude protein of two winter wheat varieties susceptible to obligate pathogens were observed. Finally, it highlighted that the response of yield, TKW, and crude protein to fungicides could vary among susceptible varieties, emphasising that the contribution of fungicides to yield achievements is closely linked to the specific reaction of each variety to pathogen infection and environmental conditions rather than solely the disease level. 

The integrated pest management (IPM) regime urges the reduction of fungicide application, and, consequently, newly developed varieties should express satisfactory disease resistance in addition to good yield and quality performances. However, there is still a lack of knowledge on factors that could affect crop performances when grown under reduced cultivation inputs. In our study, yield loss of two susceptible varieties under the same level of leaf rust infection, under conventional levels of N input, and without the interference of fungicide action was not the same. In addition, we showed that a single susceptible variety could suffer the same level of yield loss regardless of the cultivation practice and under a broad range of leaf rust infection. The results of our study support previous publications reporting that the predictions of disease effects on yield will be more accurate if the effects of pathogens on the main physiological variables (i.e., radiation interception and radiation-use efficiency) are considered [9] as well as the susceptibility of varieties to the combined effect of abiotic and biotic stressors [20,30].

Photosynthesis rate is one of the physiological variables related to radiation-use efficiency. Foliar nitrogen content is reported to be one of the major determinants of photosynthesis rate, and the net photosynthesis rate at light saturation (Pmax) of healthy leaves was shown to be significantly higher in high with respect to low nitrogen treatment [47,48]. However, information on the effects of foliar diseases on the photosynthesis of wheat leaves with different nitrogen content is extremely limited. Carretero et al. [9] reported that changes in leaf nitrogen concentration do not modify the effects of leaf rust on net photosynthesis since leaf rust affects net photosynthesis mainly through non-stomatal events, such as chlorophyll reduction. Carretero et al. [9] also suspected that leaf rust affects light interception rather than radiation-use efficiency at the crop level. Independence in metabolic actions of leaf rust and foliar nitrogen content on net photosynthesis could be one of the reason why susceptible varieties could act differently under same level of pathogen pressure and N availability. 

However, we also indicated that the effects of pathogens and N availability on net photosynthesis should not be analysed individually without considering the overall yield potential and genotype stability under unfavourable environmental conditions. The total rainfall during flowering and grain filling in 2021 was less than the eight-year averages. Varieties Barbee and Zvezdana exhibited significantly different yield losses when grown under similar levels of leaf rust infection, the same climatic conditions, without a reduction of N availability, and without the impact of fungicide action. Knowing that club wheat varieties have a higher tolerance to dry weather conditions than common wheat varieties [16], it could be expected that under the same level of leaf rust infection, a low level of total rainfall during the flowering and grain-filling time did not affect the yield loss of club winter wheat Barbee likewise it did in common wheat Zvezdana. Variety Barbee experienced higher yield losses only when the DI of leaf rust reached 29.3% and when nitrogen availability was restricted. 

Additionally, Zvezdana exhibited consistent percentage yield losses across a wide range of pathogen pressures and diverse cultivation practices. It is plausible that the high-yielding potential of variety Zvezdana contributed to the stability of yield losses, which ranged from 16% to 18.6% under various levels of nitrogen availability and pathogen pressure ranging from 11.3% to 65%. However, it should be also expected that the lower tolerance of common wheat to dry climatic conditions than that of club wheat Barbee affected the yield loss of variety Zvezdana in addition to pathogen attack, making it less correlated than in variety Barbee. In previous studies, Zvezdana is reported as highly susceptible to extreme variation in climatic factors during growing season [37]. It could be expected that the susceptibility of Zvezdana to abiotic stressors resulted in higher yield loss under a much lower disease index when compared with the response of variety Barbee. Additionally, a leaf rust infection of 11% was identified as the threshold that triggers yield reduction in this variety under unfavourable growing conditions. These results complemented the findings of Jevtić et al. [49], who suspected that there might be a threshold of pathogen pressure below which a specific cultivar will not respond with significant yield loss if other requirements necessary to exhibit its yield potential are met. 

Our study also revealed that the relationship between yield losses and thousand kernel weight (TKW) losses, were not the same for the two varieties. These results are in accordance with those of Lopes et al. [50], who indicated that variety and the environment affect TKW. However, in contrast to the study of Lopes et al. [50], where TKW and yield were correlated significantly with a positive direction, the relationship between yield and TKW loss in our study had a negative direction and was significant only for variety Barbee. There are also studies where TKW, yield, and yield components had a significant negative association [5,51,52]; thus, it could be suspected that the difference in the direction of the relationship between yield/TKW and yield/TKW loss could be attributed to differences in cultivar properties, cultivation practices, and environmental factors among studies. 

Sugár et al. [52] tested the relationship between yield and TKW under different levels of N fertilisation, and a negative association between TKW and yield was observed in all trials where N fertilisation was applied. In contrast to that, check trials without N fertilisation showed no significant correlation between TKW and yield. The tendency of decrement of TKW with rising levels of N input is also reported by Protić et al. [6]. In our study, yield and TKW loss were estimated under both monoculture with reduced-N low-input and conventional N input with crop rotation production systems. Under the same level of leaf rust infection and conventional increasing levels of N input, Barbee suffered a lower yield loss than Zvezdana. TKW loss (13.3%) and yield loss (4.7%) were significantly negatively correlated only in variety Barbee. Supply in conventional production systems significantly lowered yield loss only in the Barbee variety. Consequently, it could be suspected that conventional N-input fertilisation contributed to the lowering of yield loss in variety Barbee at the expense of TKW loss. The correlation between crude protein loss and TKW loss was also different in the two varieties and was significantly moderately negative only in variety Barbee. It could be suspected that higher levels of N input in conventional cultivation affected limited crude protein loss in variety Barbee, but it should be investigated in more detail in future studies.

In our study, leaf rust significantly affected crude protein loss. This is in accordance with previous investigations indicating that at the beginning of grain development, N accumulation is source-and-sink regulated, but during the grain filling, N accumulation is always limited by the source supply from vegetative tissues [53]. Consequently, N accumulation during grain filling could be affected by pathogens colonising vegetative organs. Although it has been reported that foliar fungal diseases in general affect nitrogen dynamics and carbohydrate accumulation in grain [53], our study found that fungicide application had differing effects on the relationship between yield and crude protein gains. This variation could be partly explained by the different effects of leaf rust on yield and crude protein achievements in the two varieties. Regression modelling in this study indicated that localities with different cultivation practices did not affect crude protein gain, thereby excluding the possibility that nitrogen inputs influenced fungicide treatments and, consequently, the differences in fungicide effects on nitrogen dynamics and crude protein response. Barbottin et al. [54] reported that nitrogen yield depends not only on nitrogen remobilisation but also on biotic and abiotic stresses during the grain-filling period. Consequently, it is plausible that during the grain-filling stage, Barbee and Zvezdana reacted differently to combined biotic and abiotic factors, resulting in different contributions of fungicide treatment to the association between yield and crude protein responses to fungicide application.

Knowing that the determination of genetic variability may not always explain the complexity of phenotype markers, phenotyping in plant breeding will always be an important practice. In addition, there is still a lack of knowledge on divergence in winter wheat responses to combined abiotic and biotic stressors, although it would facilitate decision-making in the breeding of varieties suitable for reduced-input production. Consequently, this study intended to investigate if there is a difference in the relationship between yield, TKW, and crude protein of club and common winter wheat varieties susceptible to obligate pathogens when cultivated under different cultivation practices. To our knowledge, the results of the study revealed, for the first time, the specificity in the response of susceptible varieties to leaf rust infection when cultivated under both monoculture with reduced N input and conventional cultivation practices under the same climatic conditions. This finding provides direction for further investigations focusing on (1) determining the threshold level of pathogen pressure that induces yield loss in susceptible varieties; (2) assessing the contribution of nitrogen fertilisation in reducing yield loss at the expense of thousand kernel weight loss; and (3) exploring the effects of combined biotic and abiotic factors during the grain-filling stage on the crude protein response to fungicide application.

## 5. Conclusions

The main conclusions that can contribute to the decision-making during the evaluation of winter wheat varieties’ performance are given below:

- Susceptible varieties, showing similar levels of leaf rust infection and without the impact of fungicide action, could exhibit significantly different yield losses when grown with the same cultivation practices and under the same climatic conditions;
- Contrary to Barbee, variety Zvezdana, which is more susceptible to unfavourable climatic conditions, suffered the same level of yield loss regardless of cultivation practice and under a broad range of leaf rust infection. This suggests that the level of pathogen pressure that triggers the susceptibility reaction is cultivar-specific and highly associated with the susceptibility of varieties to abiotic stressors, which should be investigated in future studies in more detail;
- Conventional cultivation could affect the reduction of yield loss in wheat varieties susceptible to leaf rust. In this study, conventional cultivation with 100 kg N per ha affected the reduction of yield loss in club winter wheat Barbee but not in Zvezdana;
- The correlation between TKW loss and yield loss was significantly moderately negative only for club winter wheat Barbee. It was suspected that N fertilisation affected the lowering of yield loss at the expense of TKW loss;
- The contribution of fungicide to yield enhancement is highly associated with the specific reaction of the variety to pathogen infection rather than solely the disease level itself.

## Figures and Tables

**Table 1:** Climatic conditions at Novi Sad (Vojvodina, north province of Serbia).

	2021	Aver. *	2021	Aver.	2021	Aver.	2021	Aver.	2021	Aver.	2021	Aver.
	January		February		March		April		May		June	
Temperature (°C)	3.3	1.4	5.1	4.6	6.2	8.0	9.6	13.0	16.0	17.0	23.3	21.8
Rel. hum.	84.0	85.8	78.0	80.5	68.0	71.4	71.0	65.3	66.0	70.3	58.0	69.0
Total rainfall (mm)	44.7	39.1	59.5	43.5	42.8	46.7	55.1	46.0	62.9	110.5	23.9	85.8

* Aver. indicates eight-year averages of temperature, relative humidity, and total precipitation.

**Table 2:** Regression analysis of the most influential factors on yield, TKW, crude protein, and oil content, along with the yield, disease index of leaf rust, and powdery mildew in untreated plots of winter wheat varieties Barbee and Zvezdana.

Dependent variable: yield					
Source	DF	Adj SS	Adj MS	F-Value	*p*-Value
Regression	3	156.89	52.27	110.11	0.000
Variety	1	115.90	115.902	244.14	0.000
Locality (cultivation system)	1	14.18	14.18	29.87	0.000
Variety × locality (cultivation system)	1	32.42	32.42	68.29	
Error	148	70.26	0.475		
Total	151	227.08			
R^2^ = 69.06%					
Dependent variable: TKW					
Source	DF	Adj SS	Adj MS	F-Value	*p*-Value
Regression	4	4838.96	1209.74	545.13	0.000
Leaf rust	1	7.14	7.14	3.22	0.075
Variety	1	1949.39	1949.39	878.44	0.000
Fungicide treatment	1	12.81	12.81	5.77	0.018
Variety × fungicide treatment	1	13.38	13.38	6.03	0.015
Error	148	328.44	2.22		
Total	152	5167.40			
R^2^ = 93.64%					
Dependent variable: Crude protein					
Source	DF	Adj SS	Adj MS	F-Value	*p*-Value
Regression	4	140.86	35.21	52.67	0.000
Variety	1	91.98	91.98	137.56	0.000
Locality (cultivation system)	1	20.82	20.82	31.13	0.000
Fungicide treatment	1	3.09	3.09	4.63	0.033
Variety × locality (cultivation system)	1	22.81	22.81	34.11	0.000
Error	148	98.963	0.6687		
Total	152	239.82			
R^2^ = 58.74**%**					
Dependent variable: Oil content					
Source	DF	Adj SS	Adj MS	F-Value	*p*-Value
Regression	7	6.15	0.87	151.17	0.000
Variety	1	2.56	2.56	440.85	0.000
Locality (cultivation system)	1	0.14	0.14	24.94	0.000
Variety × locality (cultivation system)	1	0.11	0.11	19.04	0.000
Error	145	0.84	0.01		
Total	152	6.99			
R^2^ = 87.95**%**					
Dependent variable: Disease index of leaf rust in untreated plots					
Source	DF	Adj SS	Adj MS	F-Value	*p*-Value
Regression	3	8601	2867.01	30.12	0.000
Variety	1	1229.9	1229.9	12.92	0.003
Locality (cultivation system)	1	5948.6	5948.6	62.49	0.000
Variety × locality (cultivation system)	1	1643.9	1643.9	17.27	0.001
Error	15	1428	95.20		
Total	18	10,029			
R^2^ = 85.76%					
Dependent variable: Disease index of powdery mildew in untreated plots					
Source	DF	Adj SS	Adj MS	F-Value	*p*-Value
Regression	1	919.62	919.62	39.99	0.000
Variety	1	919.62	919.62	39.99	0.000
Error	17	390.91	22.995		
Total	18	1310.53			
R^2^ = 70.17%					
Dependent variable: Yield in untreated plots					
Source	DF	Adj SS	Adj MS	F-Value	*p*-Value
Regression	2	12,933,148	6,466,574	31.78	0.000
DI leaf rust	1	3,922,916	3,922,916	19.28	0.000
Variety	1	12,586,749	12,586,749	61.85	0.000
Error	16	3,255,852	203,491		
Total	18	16,189,000			
R^2^ = 79.89%					

**Table 3:** Correlation of yield loss and crude protein loss with TKW loss of “club” winter wheat Barbee and “common” winter wheat Zvezdana.

	Barbee	Zvezdana
	TKW loss	TKW loss
Yield loss	r = −0.727, *p* = 0.011	r = −0.391, *p* = 0.235
Crude protein loss	r = −0.600, *p* = 0.05	r = 0.041, *p* = 0.905

**Table 4:** Correlation of crude protein response with TKW response and yield response of “club” winter wheat Barbee and “common” winter wheat Zvezdana after fungicide application.

	Barbee	Zvezdana
	Crude protein response	Crude protein response
Yield response	r = 0.088. *p* = 0.481	r = 0.695. *p* ˂ 0.001
TKW response	r = −0.367. *p* = 0.002	r = −0.075. *p* = 0.551

## Data Availability

The original contributions presented in the study are included in the article/Supplementary Materials, further inquiries can be directed to the corresponding author.

## Supplementary Materials

The following supporting information can be downloaded at https://www.mdpi.com/article/10.3390/jof10060401/s1, Figure S1: Regression of yield loss on crude protein loss of winter wheat varieties Barbee and Zvezdana; Table S1: Regression analysis of the most influencing factors on yield loss, TKW loss, and crude protein loss in winter wheat varieties Barbee and Zvezdana; Table S2: Regression analysis of the most influencing factors on yield, TKW, and crude protein responses to fungicide application in winter wheat varieties Barbee and Zvezdana.
