# Supplementary material for: Contrasting Performance of Two Winter Wheat Varieties Susceptible to Leaf Rust under Diverse Pathogen Pressure, Fungicide Application, and Cultivation Practices
Source: J Fungi (Basel). 2024 Jun 2;10(6):401. doi: 10.3390/jof10060401 (PMC11204494; doi:10.3390/jof10060401)
Supplement: Supplementary file 1 [file jof-10-00401-s001.zip › Supplementary Figure S1.pdf]

Supplementary information
for the manuscript:

Contrasting performance of two winter wheat varieties susceptible to leaf rust under diverse pathogen pressure, fungicide application, and cultivation practices

Radivoje Jevtić [1,2]*, Vesna Župunski [1], Dragan Živančev [1], Emilija Arsov [2], Sasa Mitrev [2], Ljupco Mihajlov [2], Branka Orbović [1]

1 Institute of Field and Vegetable Crops; 21000 Novi Sad, Serbia; radivoje.jevtic@ifvcns.ns.ac.rs (R.J.); vesna.zupunski@ifvcns.ns.ac.rs (V.Ž.); dragan.zivancev@ifvcns.ns.ac.rs (D.Ž.); branka.orbovic@ifvcns.ns.ac.rs (B.O.)
2 Faculty of Agriculture, Goce Delcev University, Stip; 2000 Stip, North Macedonia; emilija.arsov@ugd.edu.mk (E.A.);sasa.mitrev@ugd.edu.mk(S.M); ljupco.mihajlov@ugd.edu.mk (Lj.M.)
* Correspondence: radivoje.jevtic@ifvcns.ns.ac.rs; Tel.: +381 21 4898207

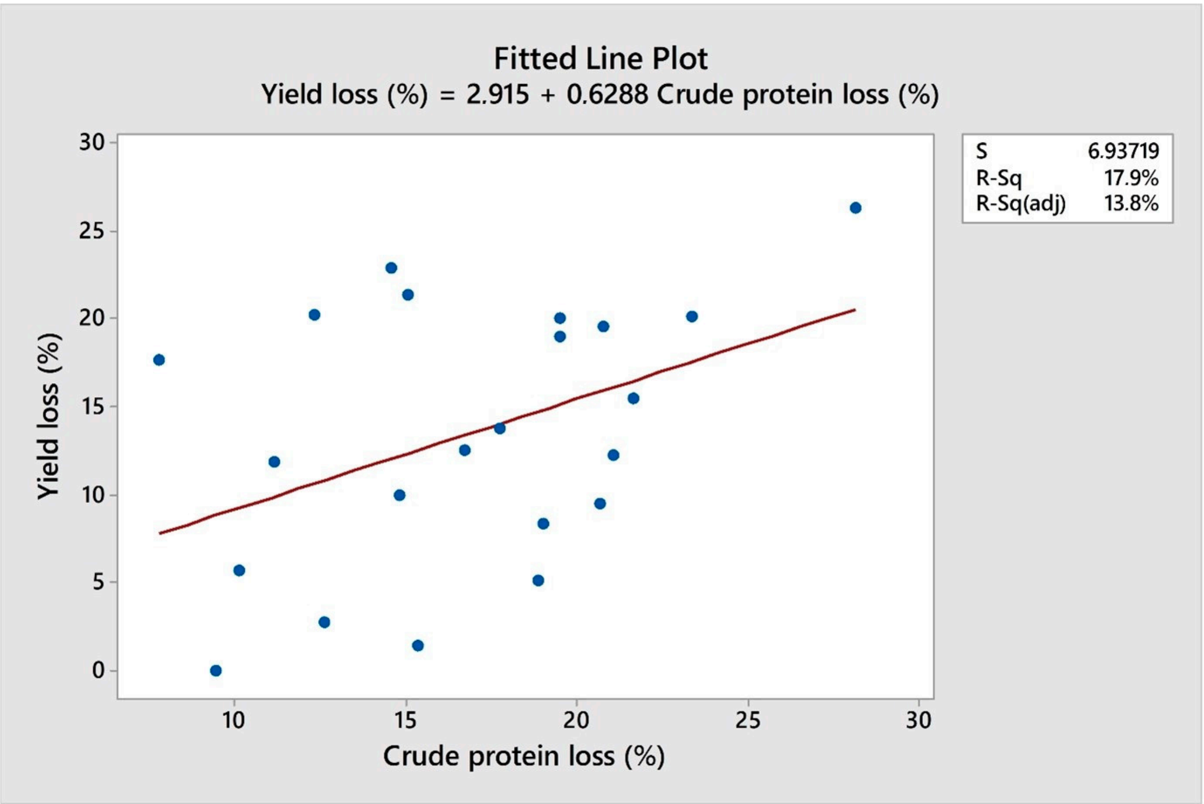

Supplementary Figure S1. Regression of yield loss on crude protein loss of winter wheat varieties Barbee and Zvezdana
