# Supplementary material for: Contrasting Performance of Two Winter Wheat Varieties Susceptible to Leaf Rust under Diverse Pathogen Pressure, Fungicide Application, and Cultivation Practices
Source: J Fungi (Basel). 2024 Jun 2;10(6):401. doi: 10.3390/jof10060401 (PMC11204494; doi:10.3390/jof10060401)
Supplement: Supplementary file 1 [file jof-10-00401-s001.zip › Supplementary Table S1.pdf]

Supplementary information
for the manuscript:

Contrasting performance of two winter wheat varieties susceptible to leaf rust under diverse pathogen pressure, fungicide application, and cultivation practices

Radivoje Jevtić [1,2]*, Vesna Župunski [1], Dragan Živančev [1], Emilija Arsov [2], Sasa Mitrev [2], Ljupco Mihajlov [2], Branka Orbović [1]

1 Institute of Field and Vegetable Crops; 21000 Novi Sad, Serbia; radivoje.jevtic@ifvcns.ns.ac.rs (R.J.); vesna.zupunski@ifvcns.ns.ac.rs (V.Ž.); dragan.zivancev@ifvcns.ns.ac.rs (D.Ž.); branka.orbovic@ifvcns.ns.ac.rs (B.O.)
2 Faculty of Agriculture, Goce Delcev University, Stip; 2000 Stip, North Macedonia; emilija.arsov@ugd.edu.mk (E.A.);sasa.mitrev@ugd.edu.mk(S.M); ljupco.mihajlov@ugd.edu.mk (Lj.M.)
* Correspondence: radivoje.jevtic@ifvcns.ns.ac.rs; Tel.: +381 21 4898207

Supplementary Table S1. Regression analysis of the most influencing factors on yield loss, TKW loss and crude protein loss in winter wheat varieties Barbee and Zvezdana

**Depenedent variable: yield loss**					
Source	DF	Adj SS	Adj MS	F-Value	P-Value
Regression	3	820.00	273.333	13.97	0.000
Variety	1	148.74	148.745	7.60	0.013
Locality (cultivation system)	1	363.94	363.941	18.60	0.000
Variety*Locality (cultivation system)	1	183.32	183.320	9.37	0.007
Error	18	352.22	19.568		
Total	21	1172.22			
**Depenedent variable: TKW loss**					
Source	DF	Adj SS	Adj MS	F-Value	P-Value
Regression	3	107.147	35.716	2.28	0.114
Variety	1	1.312	1.312	0.08	0.775
Locality (cultivation system)	1	3.355	3.355	0.21	0.649
Variety*Locality (cultivation system)	1	101.041	101.041	6.45	0.021
Error	18	281.818	15.657		
Total	21	388.965			
**Depenedent variable: crude protein loss**					
Source	DF	Adj SS	Adj MS	F-Value	P-Value
Regression	1	53.950	53.9503	2.26	0.148
Leaf rust	1	53.950	53.9503	2.26	0.148
Error	20	476.499	23.8250		
Total	21	530.449			
