# Supplementary material for: Contrasting Performance of Two Winter Wheat Varieties Susceptible to Leaf Rust under Diverse Pathogen Pressure, Fungicide Application, and Cultivation Practices
Source: J Fungi (Basel). 2024 Jun 2;10(6):401. doi: 10.3390/jof10060401 (PMC11204494; doi:10.3390/jof10060401)
Supplement: Supplementary file 1 [file jof-10-00401-s001.zip › Supplementary Table S2.pdf]

Supplementary information
for the manuscript:

Contrasting performance of two winter wheat varieties susceptible to leaf rust under diverse pathogen pressure, fungicide application, and cultivation practices

Radivoje Jevtić [1,2]*, Vesna Župunski [1], Dragan Živančev [1], Emilija Arsov [2], Sasa Mitrev [2], Ljupco Mihajlov [2], Branka Orbović [1]

1 Institute of Field and Vegetable Crops; 21000 Novi Sad, Serbia; radivoje.jevtic@ifvcns.ns.ac.rs (R.J.); vesna.zupunski@ifvcns.ns.ac.rs (V.Ž.); dragan.zivancev@ifvcns.ns.ac.rs (D.Ž.); branka.orbovic@ifvcns.ns.ac.rs (B.O.)

2 Faculty of Agriculture, Goce Delcev University, Stip; 2000 Stip, North Macedonia; emilija.arsov@ugd.edu.mk (E.A.);sasa.mitrev@ugd.edu.mk(S.M); ljupco.mihajlov@ugd.edu.mk (Lj.M.)

* Correspondence: radivoje.jevtic@ifvcns.ns.ac.rs; Tel.: +381 21 4898207

Supplementary Table S2. Regression analysis of the most influencing factors on yield, TKW, and crude protein responses to fungicide application in winter wheat varieties Barbee and Zvezdana

**Depenedent variable: yield response to fungicide treatment**					
Source	DF	Adj SS	Adj MS	F-Value	P-Value
Regression	18	2115,8	117,55	2,29	0.005
Leaf rust*Treatment	17	1895,0	111,47	2,17	0.009
Variety*Locality (cultivation system)	1	739,9	739,93	14,38	0.000
Error	112	5761,6	51,44		
Total	130	7877,4			
**Depenedent variable: TKW response to fungicide treatment**					
Source	DF	Adj SS	Adj MS	F-Value	P-Value
Regression	20	1152,82	57,64	3,65	0.000
Variety	1	347,04	347,04	21,95	0.000
Cultivation system	1	178,58	178,58	11,30	0.001
Leaf rust*Treatment	17	405,53	23,85	1,51	0.104
Variety*Locality (cultivation system)	1	34,99	34,99	2,21	0.140
Error	111	1754,92	15,81		
Total	131	2907,74			
**Depenedent variable: crude protein response to fungicide treatment**					
Source	DF	Adj SS	Adj MS	F-Value	P-Value
Regression	1	199,3	199,31	3,42	0.067
Leaf rust	1	199,3	199,31	3,42	0.067
Error	130	7576,4	58,28		
Total	131	7775,7			
